# Kinetic Energy of Throughfall in Subtropical Forests of SE China – Effects of Tree Canopy Structure, Functional Traits, and Biodiversity

**DOI:** 10.1371/journal.pone.0049618

**Published:** 2013-02-15

**Authors:** Christian Geißler, Karin Nadrowski, Peter Kühn, Martin Baruffol, Helge Bruelheide, Bernhard Schmid, Thomas Scholten

**Affiliations:** 1 Institute of Geography, University of Tübingen, Tübingen, Germany; 2 Special Botany and Functional Ecology, University of Leipzig, Leipzig, Germany; 3 Institute of Evolutionary Biology and Environmental Studies, University of Zurich, Zurich, Switzerland; 4 Institute of Biology/Geobotany and Botanical Garden, University of Halle, Halle, Germany; The Ohio State University, United States of America

## Abstract

Throughfall kinetic energy (TKE) plays an important role in soil erosion in forests. We studied TKE as a function of biodiversity, functional diversity as well as structural stand variables in a secondary subtropical broad-leaved forest in the Gutianshan National Nature Reserve (GNNR) in south-east China, a biodiversity hotspot in the northern hemisphere with more than 250 woody species present. Using a mixed model approach we could identify significant effects of all these variables on TKE: TKE increased with rarefied tree species richness and decreased with increasing proportion of needle-leaved species and increasing leaf area index (LAI). Furthermore, for average rainfall amounts TKE was decreasing with tree canopy height whereas for high rainfall amounts this was not the case. The spatial pattern of throughfall was stable across several rain events. The temporal variation of TKE decreased with rainfall intensity and increased with tree diversity. Our results show that more diverse forest stands over the season have to cope with higher cumulative raindrop energy than less diverse stands. However, the kinetic energy (KE) of one single raindrop is less predictable in diverse stands since the variability in KE is higher. This paper is the first to contribute to the understanding of the ecosystem function of soil erosion prevention in diverse subtropical forests.

## Introduction

### Biodiversity, ecosystem functioning and soil erosion

The term “Biodiversity and Ecosystem Functioning” (BEF) emerged in the beginning of the 1990ies due to growing concern about a global loss of biodiversity [1]. The basic question in functional biodiversity research is if and how biodiversity affects various ecosystem functions and services. Especially in forest ecosystems, regulating and supporting services like primary production, nutrient cycling, species conservation, soil formation, climate regulation and the prevention of soil erosion are of major interest [2], [3]. Some of these functions have already been shown to respond to gradients of biodiversity in the tree layer [4].

Soil erosion and its prevention is a central topic in the subtropics, in particular in SE China [5], [6]. Here, highly erosive rainfall causes severe and continuous soil losses, which can cause enormous economic costs [7]–[9]. Forest ecosystems may prevent erosion by water via three main mechanisms [10]–[12]:

1. interception of raindrops by leaves and branches;
2. high infiltration rates of forest soils due to low bulk densities and high pore volumes;
3. plant root systems securing the soil.

Although soil erosion is generally reduced under forests [13], high sediment loads of rivers from forested catchments, especially in subtropical regions with high rainfall intensities, are often reported [14], [15]. It can be assumed that mechanisms controlling soil erosion under forest are dynamic in space. In particular, it is conceivable that mechanism (1) can increase kinetic energy (KE) of raindrops on the soil surface due to leaf arrangements channeling small drops into larger ones [16]. A substantial increase in KE with canopy height has been found in monocultures such as beech forests in New Zealand [17], acacia forests in Indonesia [18], tropical rain forests in Colombia [19] and in Brazil [20], forest plantations in Japan [21] and secondary subtropical forests in China [22].

Variables considered to describe the amount and variation of KE in forests or throughfall redistribution under forest in general mainly refer to the characteristics of the rainfall event [21], [23], the age or height of the forest studied [24], [25], total plant cover or leaf area index (LAI) [26] and to leaf traits [21], [27].

A potentially important factor neglected so far is tree diversity, even though the relations between erosion processes and biodiversity are of great interest [28]. Although the general assumption is that biodiversity reduces soil erosion via more intensive rooting patterns (mechanism 3, [28]), empirical evidence is lacking. [29] suggested that a highly structured, diverse ground cover is the basis for soil erosion control on high mountain slopes. To our knowledge, this paper is the first addressing the role of mechanism (1), throughfall kinetic energy (TKE), on soil erosion in diverse subtropical forests.

### Increasing biodiversity reduces total TKE (hypothesis H1)

Reviews of biodiversity effects on ecosystem functioning in grassland ecosystems [30] as well as in forest ecosystems (e.g. [4]) have shown that biodiversity can increase stand level biomass, structural richness, and richness of associated plant and animal groups. Plots with increased species richness should consequently be able to intercept more rainfall than less rich ones resulting in a reduced throughfall amount. Besides that, drops are supposed to have reduced and strongly varying falling heights through structural richness and a high stand level biomass in different heights. Further, leaf-trait diversity may also affect TKE. These circumstances may result in a reduced total TKE and/or an enhanced variability of TKE in diverse forest stands for any given rainfall event.

### Higher crown openness results in a lower TKE (hypothesis H2)

Basic measures for crown openness within a specific forest stand or a specific position within a forest stand can be expressed through estimating coverage values of the different tree layers present in the canopy or LAI. LAI is of major interest when considering throughfall amount and properties. It quantifies effects of canopy thickness, leaf and branch count. However, LAI does not account for possible heterogeneity in horizontal leaf area distribution, which may also be affected by tree diversity.

Generally, throughfall amount decreases with increasing LAI as interception is enhanced at higher LAI values [23], [26], [31], [32]. In an indoor-experiment in Japan decreasing canopy thickness resulted in a higher throughfall amount and TKE, as interception is decreased as well as the re-interception probability for large throughfall drops produced by the canopy via confluence of small drops [33]. Assuming re-interception to be more important than concentration via confluence, a higher LAI therefore should result in a reduced TKE.

### Specific species and leaf traits substantially influence TKE (hypothesis H3)

Leaf traits are supposed to have a substantial effect on throughfall properties. Specifically, needles may generate different throughfall drops than broad leaves. Several drip mechanisms like “launch drip”, “tipping bucket drip”, “induced drip”, “reservoir drip” and “needle drip” are reported in the literature [34]. As the storage capacity of needles is much less than for broad leaves, the average drop size under coniferous species is generally smaller than for deciduous or evergreen species [27], [35]. Besides the distinction between broad leaves and needles, leaf area may determine the amount of water potentially to be stored on it and the size of drops subsequently released by confluence of smaller throughfall drops. Bigger leaves should be able to intercept more water and therefore produce bigger throughfall drops than smaller leaves.

### Rainfall event characteristics predefine TKE and its variability (hypothesis H4)

Rainfall event characteristics like rainfall amount, rainfall intensity and wind speed during a given rainfall event strongly influence throughfall properties. Especially events that cause a vibration of the canopy influence throughfall characteristics [35]. Tree crowns that vibrate because of the impact of large raindrops [36], intense rainfall [32], [37] or high wind speed [37] generally have a reduced storage capacity on their surfaces (leaves, branches). Therefore drops generated under conditions of canopy vibration are generally smaller (and therefore less erosive) and interception loss is strongly reduced [23]. Consequently, increased rainfall intensity and higher wind speed should decrease the variability of TKE.

We test these hypotheses with field observations carried out in a high diversity forest ecosystem in subtropical SE China.

## Materials and Methods

### Study area

This study was conducted in the Gutianshan National Nature Reserve (GNNR), Zhejiang Province, P.R. China. An entrance permit to the nature reserve was obtained by the administration of the GNNR (Zhejiang Province, China).

The GNNR is located at N 29°14.657′and E 118°06.805′. The elevation ranges from 320–910 m above sea level. The soils are predominantly Cambisols [38] developed on granite with a saprolite cover of varying thickness. The climate at the GNNR is typical of subtropical monsoon regions with an annual average temperature of 15.3°C and a mean annual rainfall of 1964 mm [39]. The secondary forest in the GNNR is extraordinary rich in species [39], [40].

In the framework of BEF-China, a multidisciplinary research unit focusing on the relationship between biodiversity and ecosystem functioning, 27 Comparative Study Plots (CSPs) of 900 m^2^ in size (30×30 m) were established in the GNNR, stratified by successional stages and selected to represent low, medium and high levels of tree species richness (for details of plot establishment, see [40]).

### Rainfall KE, TKE and other meteorological data

Rainfall KE was measured using calibrated splash cups [41] positioned next to an automatic weather station in the center of Gutianshan NNR. TKE was measured with the same device 1 m above the ground. As this study focuses solely on the influence of the tree and shrub layer the splash cups had to be positioned above the herb layer (∼1 m). Moreover, in this height disturbance by ground living animals and the influence of the inclination of the soil surface is supposed to be minimized.

The major advantage of splash cups is their easy handling and the high number of replications that can be obtained. Moreover, they are able to measure the whole event rather than a short time span only. The splash cups used in this study had a diameter of 4.6 cm and a surface of 16.62 cm^2^. The loss of sand from the cups was converted into KE per area using a calibration function. For further information about measurement procedure and calibration function see [22], [41]. Rainfall amount, rainfall intensity and wind speed were recorded by an automatic weather station located in the center of GNNR, using the Vaisala WXT520 Sensor. Its measurement principle is a piezo-electric surface, which is sensitive to raindrop impact. During every rainfall event, five splash cups were exposed to rainfall close to the automatic weather station.

### Tree-canopy and rainfall-event related variables

In every study plot, vegetation characteristics were recorded that described the diversity of tree and shrub species, crown openness and mean leaf traits of the forest community (see Appendix S1).

Event-related variables include rainfall amount, intensity and KE as well as wind speed during the events. Individual variables were tested for collinearity before setting up the models. They were sorted by five groups in the order of their expected impact, namely event characteristics, age/height of the vegetation, diversity measures, crown openness and leaf traits:

1. *Event characteristics*: Using an automatic weather station, rainfall amount, rainfall intensity as well as wind speed were measured in 5-min intervals for all events. Rainfall KE was measured using splash cups (see above).
2. *Age-height*: Using three different tree canopy layers, we calculated a weighted average of canopy height by using tree layer cover as weight. As an estimation of age we weighed the abundance of trees >10 cm in diameter at breast height (dbh) by size and number.
3. *Diversity*: We quantified tree canopy diversity using species richness of individuals >1 m height. Moreover, rarefied species richness was calculated using rarefaction curves [42]. This method allows comparing species richness of sites with different numbers of tree individuals. In the present study adult richness per 100 individuals was calculated [40]. As an alternative measure for biodiversity we used the functional diversity of tree leaf traits. We included specific leaf area, leaf size as well as presence/absence of dentate, pinnate, or needle leaves of all individuals found in the plots. Based on tree species abundance in the plots, we calculated functional identity and functional dispersion of each plot [44].
4. *Crown openness*: LAI as a measure of crown openness was assessed using hemispherical photographs. Photographs were taken in each of the 9 subplots (10×10 m) using a Canon EOS 350 with a Sigma 8 mm fisheye-lens mounted. To ensure the photographs being taken horizontally a monopod was used. As a further measure of crown openness, the estimated coverage values of the tree layers were included in the candidate models.
5. *Leaf traits*: A measure of “leaf trait functional identity” was calculated for each plot as species-abundance-weighted mean of a leaf trait. We considered the weighted mean of specific leaf area and leaf size as numeric traits and coded presence/absence of dentate, pinnate, or needle leaves as 0 and 1.

### Sampling design

The sampling design of the splash cup measurements consisted of a plot-based approach with 15 splash cups per plot. From the 27 CSPs set up in BEF China, 10 plots of contrasting biodiversity were selected for this study in order to maximize the biodiversity gradient (see Appendix S1). The plots used have an average distance of 2 km (maximum distance  = 3.9 km) from the reference station in the center of the GNNR. The splash cups were positioned randomly in each CSP according to a 1 m wide grid resulting in 900 possible positions. Consequently, the splash cups had a minimum distance of 1 m to each other. When a tree or any other obstacle was encountered at the envisaged position, the splash cup was positioned at the next possible position. The splash cups were numbered 1 to 15 and the positions remained constant during the events measured.

### Statistical analysis

The effect of the above mentioned variables on TKE and its variability was tested using mixed-effects models (e.g. [43]). Analyses were performed using event and plot, as well as the positions of the splash cups within the plots, as random factors. Due to the absence of nesting between rainfall events and plots/positions (these two nested), we used mixed models that allowed crossed random factors. With this approach we could quantify the variance components of the splash cup positions within plots across the different rainfall events. All our candidate models included one variable from each of the five predictor groups introduced above. Starting with the characteristics of the rainfall event, we used backwards selection to identify the most important variable in each of the groups, if any. Since all models had the same random variable structure, we fitted the models using maximum likelihood and compared them based on AIC. The resulting best model was then refitted using Restricted Maximum Likelihood [43]. We used a similar approach for the variability of KE, where the standard deviation for the 15 individual splash cup positions within plot and event was calculated. Since the lowest spatial level for variability of TKE was the plot level, splash cup positions were not included in this model. All analyses were performed using R 2.12 [45] together with the packages “lme4” [46] and “multcomp” [47]. Functional trait measures were calculated using the “FD” package [44].

## Results

### Relation between KE of open field rainfall and TKE

**Figure 1:**
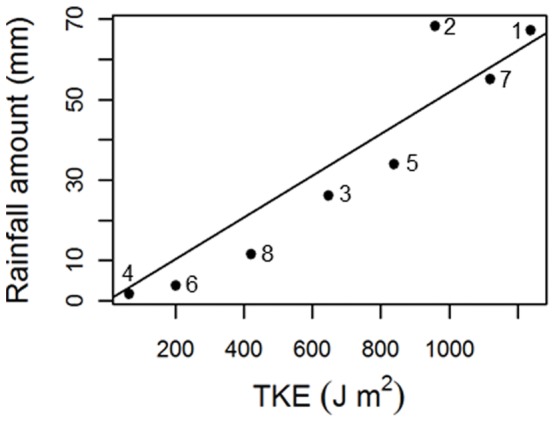
Relationship between rainfall amount and throughfall kinetic energy (black dots  =  events, numbers  =  event numbers mentioned in Table 1)**.**

The rainfall amounts for the measured events ranged from 1.8–68.3 mm, while the maximum intensities ranged between 1.2 mm/h and 55.7 mm/h (Table 1). The lowest rainfall KE was measured during event 4 (13.1 J m-2) while the highest rainfall KE was measured during event 1 (717.0 J m-2) with an average rainfall KE of 297.2 J m-2 over all events measured. TKE ranged from 62.7 J m-2 for event 4 and 1237 J m-2 for event 1 (average 685.1 J m-2). The data show a linear increase of TKE for increasing rainfall amounts over the events measured (Figure 1). On the average, TKE is 2.3 times higher than adjacent rainfall KE.

**Table 1:** Characteristics of the rainfall events measured.

Rainfall event	Rainfall amount (mm)	Peak rainfall intensity (mm h^−1^)	Kinetic energy of rainfall (J m^2^)	average kinetic energy of throughfall (J m^2^)
1	67.3	55.7	717.0	1237.0
2	68.3	33.8	701.5	958.3
3	26.2	9.0	137.5	646.3
4	1.8	1.2	13.1	62.7
5	34.0	18.7	273.2	837.9
6	3.8	5.2	26.3	199.8
7	55.1	33.0	413.2	1119.3
8	11.6	4.6	96.3	419.5
average	33.5	20.1	297.3	685.1

### Relation between TKE, forest stand variables and biodiversity

**Figure 2:**
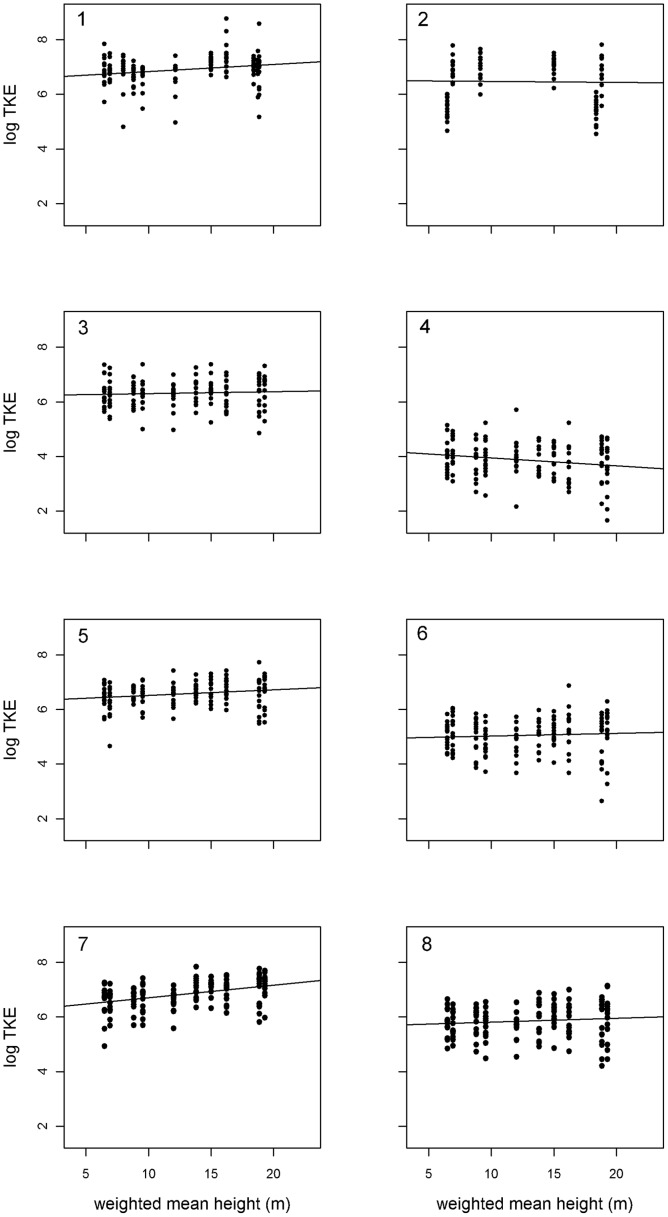
Relation between log TKE and weighted mean height of the tree layers for rainfall events 1 to 8. Black dots represent the study plots. See Table 1 for the properties of the precipitation events.

Our data showed that although the relation of TKE to rainfall amount was modulated by tree canopy height, the effect of rainfall amount dominates the magnitude of raindrop energy (Table 2, Figure 2). Increasing rainfall amount produced overall higher raindrop energies, but the events with the low rainfall amount (e.g. Figure 2, No. 4) the TKE slightly decreased with canopy height, while for higher rainfall the effect of canopy height approached zero and had a small but positive effect for the highest rainfall amounts (e.g. Figure 2, No. 1). Rainfall intensity, rainfall KE and wind speed (group of “event characteristics”) all did not improve model quality and were therefore omitted.

**Table 2:** Effects influencing throughfall kinetic energy (TKE).

	Estimate	Std. Error	z value	Pr(>|z|)	sig.
(Intercept)	5.919	0.206	28.678	<2e-16	***
rainfall amount	0.801	0.196	4.078	4.54E-05	***
weighted mean height of the vegetation	−0.132	0.063	−2.104	0.035	*
biodiversity (rarefy 100)	0.127	0.064	1.968	0.049	*
coniferousness (proportion of coniferous species)	−0.117	0.055	−2.127	0.033	*
LAI	−0.114	0.058	−1.975	0.048	*
rainfall amount: weighted mean height	0.100	0.014	7.099	1.25E-12	***

Results of the simplified mixed effects model. Fixed factors in the model were the predictors and their interactions as shown in the table. In addition, the two crossed random factors were position of splash cup nested in plot and rainfall event.

Signif. codes: 0 ‘***’ 0.001 ‘**’ 0.01 ‘*’ 0.05 ‘.’ 0.1 ‘ ’ 1.

(Univariate p values reported).

Among the “diversity measures” rarefied species richness significantly increased TKE. However, functional leaf trait diversity appeared to have no significant effect and did not enter the final model. LAI as a variable describing “crown openness” had a significant negative effect on TKE. The higher the LAI, the lower is TKE. Tree layer cover and non-rarified species richness did not improve model quality.

Among “leaf traits”, the proportion of coniferous species in a given plot had a significant negative impact on TKE. Plots with a higher proportion of coniferous species in the tree layer generally receive less TKE. Variables not mentioned further here did not improve model quality.

### Variability of TKE related to event characteristics and biodiversity

**Figure 3:**
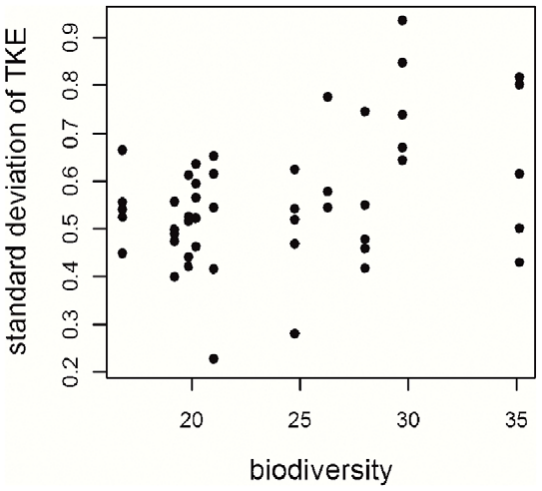
Relation between the standard deviation of TKE and biodiversity. Each dot represents a study plot.

The standard deviation of TKE was significantly affected by rainfall intensity and biodiversity (Table 3). Rainfall intensity decreased the variability of throughfall, while rarefied species richness increased the variability (Figure 3). Leaf area, LAI, weighted mean height and functional diversity did not enter the final model, since their inclusion reduced model quality.

**Table 3:** Effects influencing the standard deviation of throughfall kinetic energy (TKE).

	Estimate	Std. Error	z value	Pr(>|z|)	sig.
(Intercept)	0.533	0.026	20.531	<2e-16	***
rainfall intensity	−0.087	0.015	−5.745	9.17E-09	***
biodiversity (rarefy 100)	0.050	0.024	2.116	0.034	*

Results of the simplified mixed effects model. Fixed factors in the model were rainfall intensity and rarified richness of woody species. In addition, the two crossed random factors were plot and rainfall event.

Signif. codes: 0 ‘***’ 0.001 ‘**’ 0.01 ‘*’ 0.05 ‘.’ 0.1 ‘ ’ 1.

(Univariate p values reported).

## Discussion

In this study we did not only demonstrate that biotic variables influenced the average TKE, but also its variability. We also showed that the effects of rainfall event characteristics were modulated by tree diversity and canopy structure.

### Factors influencing throughfall kinetic energy

The significant interaction between rainfall amount and tree canopy height showed that there is a different relation between height of the vegetation and TKE for different rainfall amounts. Moreover, this means that with increasing rainfall amount the effect of canopy height is changing direction from slightly negative to slightly positive. This suggests that in older, structurally more complex forests rainfall events need to be of a certain amount until TKE can reach higher values.

We might need additional variables describing canopy structure to explain this counterintuitive relationship of decreasing TKE with increasing canopy height. This conclusion is supported by the stable spatial pattern of KE quantified by the variance component of the splash cup position. This shows that different positions within the plot received similar KE across rainfall events, which could not be explained by the variables we tested. These variables seem to modulate TKE especially in conditions of relatively low amounts of rainfall, while canopy height retains the expected positive relation to KE for high amounts of rainfall. For further studies we plan to apply laser scanning techniques to quantify additional spatial properties of the canopy apart from canopy height.

Besides height, identity of the tree traits, crown openness and biodiversity also influenced TKE significantly.

However, in contrast to our first hypothesis, raindrop energy tended to be higher rather than lower in more diverse forests. Since this is an observational study, we cannot rule out that the biodiversity gradient is correlated to other plot-level characteristics, such as soil conditions, successional age and altitude [40]. However, the plots in this study have been selected in a stratified way to maximize the range of tree species richness within one successional age class. Since canopy height is an indicator of stand age, we conclude that the biodiversity effect exists above and beyond the successional age effect.

It has been suggested that increased richness and biomass in diverse forests results in a highly structured groundcover (litter, herbs) with high cover rates, thereby absorbing a considerable amount of KE produced by the canopy layers. However, a direct link between herb- and tree-layer diversity could not be found in the study area [48]. Nevertheless, regarding the soil itself, [28] showed that soils in highly diverse forests are characterized by a higher rooting density (mechanism 3, cf. Introduction). This may, at least partly, compensate highly erosive throughfall reaching the soil surface.

Crown openness also had a direct impact on TKE by increasing KE with decreasing LAI. Mainly, two mechanisms could play a role in the reduction of TKE with increasing LAI. At first, forest stands with higher LAI generally tend to intercept more rainfall (e.g. [26], [49], [50]) resulting in a reduction of throughfall amount which negatively affects TKE. Secondly, drop size and therefore KE could be affected by re-interception [33]. Falling drops in thicker canopies or forests with a high coverage of several tree layers are more likely to be re-intercepted and split by lower parts of the canopy which can have a negative effect on TKE [18]. Therefore, multi-layered canopies with a larger vertical extension are supposed to generate less throughfall. Regarding branching structures further studies on their impact in relation to biodiversity or TKE (e.g. [51]) may be helpful in predicting how the structure of the canopy volume influences raindrop redistribution and size. Nevertheless, these structures seem to promote (regardless of season) a stable spatial pattern of TKEor throughfall amount [23]. Splash cups measuring at the same location within a plot but at different points in time were correlated and model quality was substantially improved when incorporating the specific positions of the splash cups within a plot. Concerning accurate drop sizes and numbers the application of laser disdrometers is suggested. By the application of at least two instruments it would be possible to calculate a budget of drop count, size and velocity between forest and open field However, due to technical and financial demands only exemplary studies in more homogenous forests are reasonable and realizable as the number of possible replications is limited.

Beside crown openness and diversity of the plant community expressed as rarefied species number, also the identity of the tree traits contributed to explaining variation in TKE. In our study, a higher proportion of needle-leaved tree species reduced TKE within a plot. As needle leaved species have a much lower leaf surface area than broadleaved species, the maximum drop size to be released is much lower. This is mostly due to a different drip mechanism [34], [35] and a lower maximum storage capacity per leaf. However, since we also included mean leaf area in the candidate models, which could not explain as much variation, there must be further mechanisms beyond leaf area alone to needles that prevents drops from growing larger.

### Factors influencing the variability of throughfall

In contrast to several factors explaining average TKE, its variability is controlled by two factors only. Furthermore, the effect sizes of these two factors are comparable and do not differ by one order of magnitude as in the analysis on mean TKE. Concerning the variability of TKE, event characteristics and diversity measures independently explained a significant amount of variation in the variance of TKE in the plots. This variance of raindrop energy can be interpreted in two ways: (1) as a measure of raindrop size diversity and (2) as the heterogeneity of the spatial distribution of TKE in total. A high variance means that very small as well as very large raindrops have reached the splash cups, while a low variance indicates more uniform raindrops. Rain-shadow positions in a dense diverse canopy could also affect the variability in keeping rainfall away from certain positions. In contrast to the previous analysis, rainfall intensity affected this variance more than rainfall amount, which was the best measure for the variability of raindrop energy below canopies.

According to our data, increasing rainfall intensity as well as increasing rainfall amount led to a decreased variability of TKE. As described above, KE under forest was closely connected to rainfall amount interacting with weighted mean height. Moreover, further related variables (e.g. biodiversity, LAI, proportion of coniferous species) were responsible for re-distributing rainfall by temporary canopy storage. Nevertheless, concerning higher rainfall intensities storage on leaves is reduced because of two reasons reported in literature resulting in canopy vibration: (1) frequent impact of large drops and (2) high wind speeds. Both are often associated with high rainfall intensities (e.g. [35]). In our study however, we could not prove the influence of (2) wind speed. Nevertheless we can say: for a given position within a forest an increase in rainfall intensity results in the condition that the position will be less likely in a rain-shadow. Thereby spatial variability of TKE is reduced.

The significant positive relationship between variability of TKE and biodiversity is reasonable as both the variability of TKE and biodiversity are measures of heterogeneity. Especially biodiversity better describes this structural heterogeneity than other measures used in this analysis like e.g. LAI, which does not account for these. In highly diverse forests the number of potential drip points is enlarged as diverse forests are geometrically more complex compared to less diverse forests [4], [23]. With increasing diversity of a forest stand, also the variability of TKE increases substantially. Therefore biodiversity can be seen as an important factor enlarging raindrop energy variability. This also means that there are highly energetic drops produced under diverse canopy conditions. If this is true, highly diverse forests have to deal with higher energetic drops arriving below the tree and shrub layers. Future research in diverse forests including herbs, other near-surface vegetation and litter cover needs to show how these complex ecosystems cope with this situation. Furthermore, as it has been demonstrated by other studies that both throughfall volume and TKE are species-specific [22], [23], [27], [35], a higher number of randomly assembled species in a given plot should result in a more heterogeneous pattern of throughfall amount and TKE. As a corollary, it is conceivable that the positive effect of biodiversity on variability in TKE leads to increased micro-environmental heterogeneity, which in turn might promote biodiversity of e.g. soil organisms and processes.

Summing up we can state that the variability of TKE is highest during events with low peak rainfall intensities and in plots with high tree species richness.

## Conclusions

This study is the first to reveal mechanisms and processes of soil erosion prevention related to biodiversity acting in quasi-natural species rich forests. The calibrated splash cups used in this study across a large number of plots turned out to be a feasible but also highly effective and reliable tool to measure the amount and variability of TKE. It could be shown that TKE in forests was higher than in the open field which was mainly caused by a redistribution of throughfall volume in forests and altered drop sizes.

Rainfall amount and several biotic factors showed to be important factors explaining and influencing TKE in forests. Here, we could demonstrate that, especially under conditions of high rainfall amounts, taller (older) forests face a higher risk of splash-induced erosion than shorter (younger) forests. Apart from canopy height, other biotic factors influence TKE in forests, in our case LAI, biodiversity (tree species richness) and the proportion of needle-leaved species. In contrast to our first hypothesis (H1), TKE increased instead of decreased with biodiversity in the plots. As we focused only on the tree and shrub layer when describing the biodiversity of a plot the role of lower shrubs, herbs and other near-surface vegetation for TKE remains an open question and deserves further research.

In consistence with the second hypothesis (H2) higher crown openness (and therefore a lower LAI) resulted in higher TKE values. In turn a higher LAI reduced TKE and throughfall amount. The mechanism of drop retention and re-interception is therefore supposed to superimpose the mechanism of concentration of water via confluence which would result in larger drops.

Moreover – and corresponding to the third hypothesis (H3) – we showed that a higher proportion of needle leaved species within a specific forest stand reduced TKE. This shows a strong influence of canopy composition with regard to leaf traits. Nevertheless, we did not encounter a difference between deciduous and evergreen species. Here, especially phenology may play a major role.

In contrast to TKE itself the variability of TKE could be described by rainfall intensity and biodiversity, which corresponds to hypothesis 1 (H1) and hypothesis 4 (H4). These two factors seem to act independently of each other in the forest ecosystem studied. The variability of TKE was highest in highly diverse forests during rainfall events with low intensities.

## Supporting Information

Appendix S1 **Plot characteristics.**
(XLS)
